# A rare case report of late-onset phytobezoar formation following laparoscopic sleeve gastrectomy: delayed redo bariatric surgery

**DOI:** 10.1186/s12893-021-01254-8

**Published:** 2021-05-22

**Authors:** Shayan Aryannezhad, Yasaman Sadeghian, Parvin Shapoori, Majid Valizadeh, Maryam Barzin

**Affiliations:** 1 Obesity Research Center, Research Institute for Endocrine Sciences, Shahid Beheshti University of Medical Sciences, Tehran, Iran; 2 Department of Surgery, Faculty of Medicine, Tehran Obesity Treatment Center, Shahed University, Tehran, Iran

**Keywords:** Bariatric surgery, Bezoars, Gastrectomy, COVID-19, Case report

## Abstract

**Background:**

Phytobezoar formation is a complication of bariatric surgery and mostly occurs after laparoscopic Roux-en-Y gastric bypass (LRYGB) operations. Here, we present an extremely rare case of late phytobezoar formation following laparoscopic sleeve gastrectomy (LSG).

**Case presentation:**

A 52-year-old woman with a body mass index (BMI) of 40.7 kg/m^2^ underwent LSG. Following persistent symptoms of nausea, vomiting, early satiety, and tremendous weight loss, endoscopy was performed, and gastric phytobezoar was detected at one-year post-operation. After endoscopic fragmentation, phytobezoar was removed by snare, and the patient later underwent redo bariatric surgery (conversion of LSG to LRYGB).

**Conclusions:**

With an increase in the number of LSG procedures performed globally, and the late-onset nature of phytobezoar formation, more cases of this complication are expected to be detected in future. Long-term postoperative follow-up alongside applying surgical methods to avoid gastric stenosis are needed to reduce the chance of phytobezoar formation in patients undergoing LSG.

## Background

Bezoars, which are intraluminal hard masses or concretions in the gastrointestinal (GI) system caused by the accumulation of indigestible ingested materials, are most commonly found in the stomach. Phytobezoars, as fiber-rich residues of vegetables and fruits, are the most common types of bezoars [1]. The etiology of phytobezoar formation is multifactorial encompassing both personal (e.g., consumption of high amounts of fibrous foods, gastric motility disorders) and anatomical (e.g., GI tract obstruction due to strictures, diverticulum, or tumors) factors [2]. The most common complications of phytobezoars are gastric ulceration and intestinal obstruction; they can also be asymptomatic or present with a variety of GI symptoms [3].

Bariatric surgery has been proposed as the best available strategy to treat morbid obesity. Laparoscopic Roux-en-Y gastric bypass (LRYGB) and laparoscopic sleeve gastrectomy (LSG) are the two most frequently performed procedures today. Currently LSG has more favorable outcomes and is considered as an effective primary bariatric surgery [4]. While generally considered to be safe, bariatric operations permanently alter the patient’s GI anatomy and, may lead to postoperative complications [5].

Moreover, the patients undergoing bariatric surgery are at the risk of the formation of phytobezoars due to reduced gastric motility, loss of pyloric function, hypoacidity, having a small gastric pouch, and most importantly, GI tract stenosis and strictures. Phytobezoars following bariatric surgery are mostly reported after LRYGB operations. LSG, preserves pyloric function, so extremely rare cases of phytobezoar formation post-LSG are mainly attributed to gastric stricture, especially in the incisura [6]. We here report a case of late phytobezoar formation after LSG, which required the patient to be re-operated.

## Case presentation

**Fig. 1:**
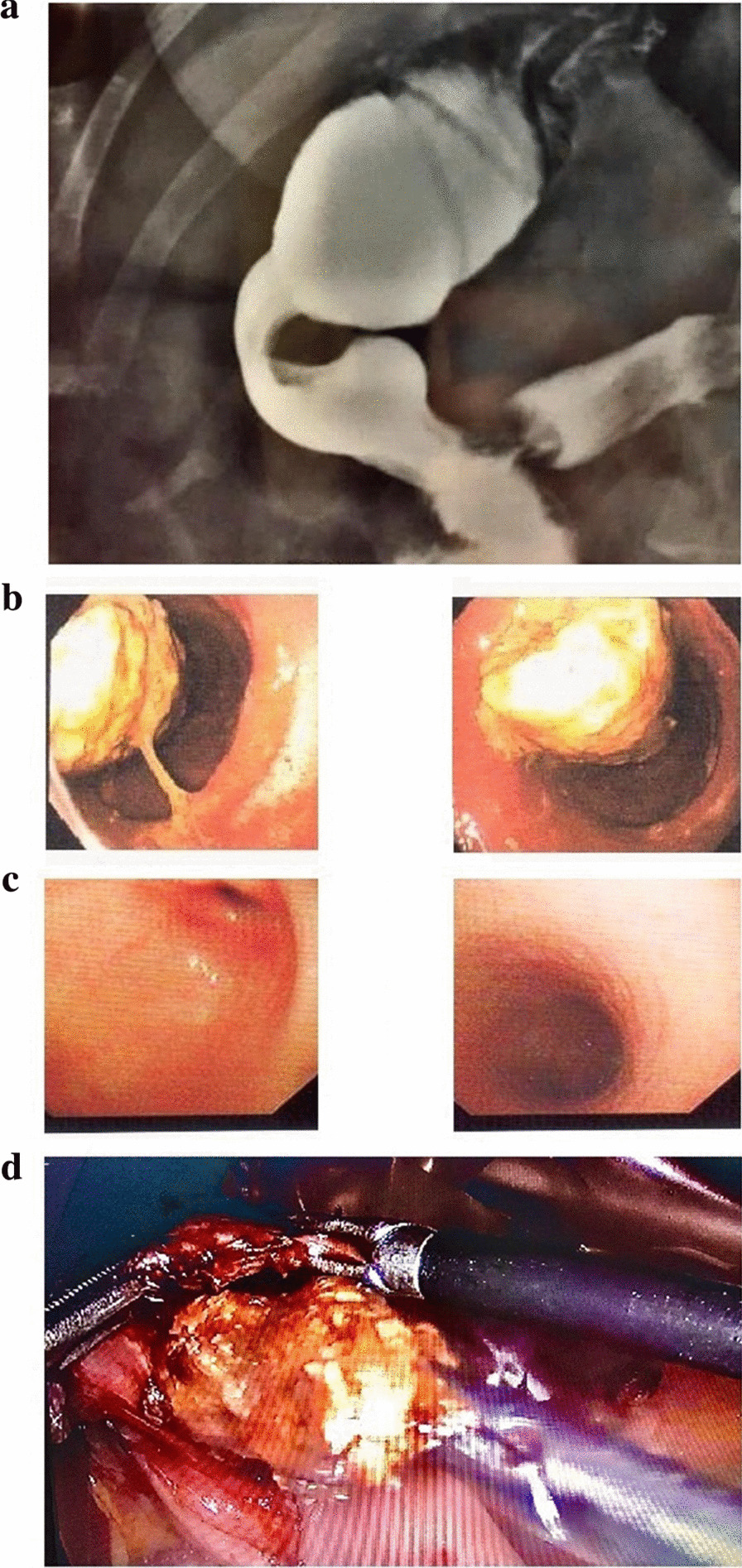
The patient’s endoscopic and radiologic images. **A** At three-month post-LSG, barium meal (upper GI series) indicated incisura stricture. **B** One year after the operation, a large gastric phytobozoar was seen in the body of the stomach. **C** Control endoscopy showed normal gastric antrum and bulb after the endoscopic removal of the phytobezoar by a snare. **D** The phytobezoar observed in the redo bariatric surgery

A 52-year-old woman with a body mass index (BMI) of 40.7 kg/m^2^, without any underlying comorbidity or history of past surgeries, was referred for LSG. Two weeks prior to the operation, endoscopy was performed, which revealed the signs of minimal gastro esophageal reflux disease (GERD) in the esophagus and stomach, as well as *Helicobacter pylori* infection. The patient received appropriate treatments for these problems. She was a participant of the Tehran Obesity Treatment Study (TOTS) [7]. LSG was performed in August 2019, followed by normal post-operation recovery and routine nutritional consult at discharge [8]. In brief, the LSG procedure was performed by excision of 80 % of the stomach. Using several stapler firings, the gastric tube was formed over a 36-F bougie and reinforced with running sutures and an omental pouch. At one-month post-operation, she experienced nausea, vomiting, and early satiety. She also had attempts for self-induced vomiting. The percentage of excess weight loss (EWL%) was 24.0 %, and BMI reduced to 36.9 kg/m^2^. Antacid therapy (Pantoprazole) was initiated. At three-month post-operation (EWL% = 63.8 %, BMI = 30.7 kg/m^2^), due to the persistence of the patient’s complaints, endoscopy was performed, which showed mild antral gastritis and GERD (grade B). She was advised to continue antacid therapy and refer for follow-up visits. Due to the persistence of her symptoms, barium meal (upper GI series) was performed, which indicated incisura stricture (Fig. 1 A). Therefore, she became a candidate for redo bariatric surgery. However, due to the Coronavirus disease (COVID-19) lockdown, she was unable to refer on time, and her follow-up nutritional assessments were not held as TOTS’s routine protocol [8]. After one year of the operation, she returned with significant weight loss (EWL% = 95.5 %, BMI = 25.7 kg/m^2^) and the continuation of previous symptoms. So, she underwent endoscopy again, which showed medium-sized sliding hiatus hernia, large gastric phytobezoar in the body of the stomach, and suspicious rotation of antrum anatomy. Upon follow-up questioning, the patient reported routine consumption of raisins, seeds, and plums in her dietary habits. Following the consumption of oral Coca-Cola® drink for three days, another endoscopy was performed. The phytobezoar’s size was reduced, and after fragmentation, it was removed by snare (Fig. 1B). Afterwards, control endoscopy showed a normal stomach without any phytobezoar (Fig. 1 C). However, her symptoms persisted, and following the previous diagnosis of incisura stricture, she underwent redo bariatric surgery (conversion of LSG to LRYGB) in November 2020 (EWL% = 98 %, BMI = 25.0 kg/m^2^). Incisura stricture and phytobezoar were observed during operation (Fig. 1D). After the surgery, the patient experienced severe bleeding from the drain site, which led to the transfusion of packed red cells. She was then stabilized, recovered, and discharged four days after the operation. She easily progressed toward consuming a regular diet. At one-month post-LRYGB, she had a good general condition and no symptoms.

## Discussion and conclusion

As the number of bariatric surgeries is increasing globally, it is important to inform surgeons and physicians about the possible complications of these surgeries. Clinicians should be aware of phytobezoar’s symptoms and signs particularly because this rare complication is prone to be neglected as it occurs late in post-operative period. Previous literature implies that bezoars happen mainly after LRYGB surgeries, but they are rare following LSG [6, 9–11]. As LSG is a bariatric procedure with a rising popularity, and phytobezoar formation can occur several years after the operation, more cases of post-LSG phytobezoar formation are expected to be reported in the future.

Prior gastrectomy has been proposed as the main risk factor of bezoar formation [12]. LRYGB delays gastric emptying and slows down the intestinal transit time, and include GI anastomosis which increases stricture risk [6]. Even though LSG procedure does not include a GI tract anastomosis, it alters gastric motility and acidity, and in combination with incisura stricture, it may exaggerate the risk of bezoar formation, which may be the case of our patient. From March 2013 to August 2019, this was the first report of phytobezoar formation among 2905 cases of LSG, 1155 cases of laparoscopic mini gastric bypass, and 169 cases of LRYGB in TOTS subjects (incidence rate: 0.02 %).

Treatment options of bezoars should be tailored to the patient’s condition. Consuming chemical solvents should always be considered as a first-line option because it is noninvasive and inexpensive. Endoscopic therapy after fragmenting the bezoar should be considered next. However, due to limitations of endoscope probe maneuvers in a sleeved stomach, even a normal endoscopic view cannot confidently guarantee bezoar removal, as happened in our case. Therefore, surgical removal should be reserved for patients with persistent symptoms, non-respondents to less invasive treatments, and those who suffer from anatomical defects (e.g., incisura stricture). In our experience, the patient underwent both chemical dissolution therapy by drinking Coca-Cola® and endoscopic fragmentation and removal by a snare, but surgery was performed as the final treatment.

Phytobezoars can appear even years after bariatric surgeries such as LSG and LRYGB. Thus, it is essential to emphasize the importance of long-term follow-up in these patients. Clinicians must bear in mind gastric stricture as a possible postoperative complication, especially in the patients presenting with recurrent phytobezoars. In order to reduce the risk of phytobezoars, the surgical team performing LSG must consider preventive strategies to avoid gastric stenosis (i.e., incisura stricture), unintentional narrowing of the stomach (tubular diameter), and helical twist of tabularized stomach.

## Data Availability

Data sharing not applicable to this article as no datasets were generated or analyzed during the current study.

## Abbreviations

LRYGB: Laparoscopic Roux-en-Y gastric bypass
LSG: Laparoscopic sleeve gastrectomy
BMI: Body mass index
GI: Gastrointestinal
GERD: Gastro esophageal reflux disease
EWL: Excess weight loss
